# Effects of individualized resistance training prescription with heart rate variability on muscle strength, muscle size and functional performance in older women

**DOI:** 10.3389/fphys.2024.1472702

**Published:** 2024-12-17

**Authors:** Diego Bittencourt, Ramon Martins de Oliveira, Deivid Gomes da Silva, João Guilherme Almeida Bergamasco, Marcelo de Castro Cesar, Daniela Godoi Jacomassi, Júlio Benvenutti Bueno de Camargo, J. Derek Kingsley, Cleiton Augusto Libardi

**Affiliations:** ^1^ MUSCULAB - Laboratory of Neuromuscular Adaptations to Resistance Training, Department of Physical Education, Federal University of São Carlos (UFSCar), São Carlos, Brazil; ^2^ Department of Medicine, Federal University of São Carlos (UFSCar), São Carlos, Brazil; ^3^ DINÂMICA - Motor Behavior Laboratory, Department of Physical Education, Federal University of São Carlos (UFSCar), São Carlos, Brazil; ^4^ Exercise Science and Exercise Physiology, School of Health Sciences, Kent State University, Kent, OH, United States

**Keywords:** autonomic nervous system, elderly, functionality, muscle hypertrophy, recovery

## Abstract

**Introduction:**

This study aimed to investigate whether individualizing autonomic recovery periods between resistance training (RT) sessions (IND) using heart rate variability (HRV), measured by the root mean square of successive R-R interval differences (RMSSD), would lead to greater and more consistent improvements in muscle strength, muscle mass, and functional performance in older women compared to a fixed recovery protocol (FIX).

**Methods:**

Twenty-one older women (age 66.0 ± 5.0 years old) were randomized into two different protocols (IND: n = 11; FIX: n = 10) and completed 7 weeks of RT. Measurements of RMSSD were performed within a five-day period to establish baseline values. The RMSSD values determined whether participants were recovered from the previous session. The assessments included muscle cross-sectional area (CSA), one-repetition maximum (1RM), peak torque (PT), rate of force development (RFD), chair stand (CS), timed up and go (TUG), 6-minutes walking (6MW), and maximum gait speed (MGS).

**Results:**

There were no significant (*P* > 0.05) group vs. time interactions. There were significant main effects of time (*P* < 0.05) for CSA, 1RM, PT, TUG, CS, 6MW, and MGS, while no significant changes were observed for RFD (*P* > 0.05).

**Conclusion:**

IND does not seem to enhance responses in muscle mass, strength, and functional performance compared FIX in healthy older women.

## Introduction

Resistance training (RT) stands as a primary non-pharmacological strategy recommended to increase muscle mass, strength, and functional performance in the older adults. Interestingly, a substantial variability in the adaptations induced by RT has been observed in older adults (Ahtiainen et al., 2016). Although factors such as baseline fitness level, genetics, and nutritional status are known determinants of individual differences in responses to RT, it has been suggested that the recovery interval between sessions also may influence some adaptations (Ahtiainen et al., 2016). For instance, a high RT strain in association with inadequate recovery periods could lead to the accumulation of fatigue and impair the repair of training-induced microtraumas, potentially resulting in a decrease in the performance of subsequent sessions, which might impact RT-induced adaptations (Smith, 2004; Xiao et al., 2012; Grandou et al., 2020). In contrast, an optimal recovery period empowers individuals to endure higher loads or execute more repetitions in subsequent workouts (i.e., volume load equals the product of sets, repetitions and load) (Chaves et al., 2024), which is associated with adaptations following RT based on the Position Stand from the National Strength and Conditioning Association (Fragala et al., 2019). Therefore, it is plausible to suggest that adequate recovery between sessions can reduce the accumulation fatigue and ensure the repair of training-induced microtraumas in older adults. This approach may ensure greater adaptation to RT and potentially reduce the significant variation in adaptations observed between subjects.

Among non-invasive tools used to quantify the accumulation of training-induced fatigue during periods of high training load, heart rate variability (HRV), particularly the root mean square of successive R-R intervals (RMSSD) parameter, a measure of parasympathetic modulation, has received considerable attention in the field of exercise science (Duking et al., 2021). The RMSSD has been adopted as a practical tool to evaluate training effectiveness, as it reflects the parasympathetic activity even over short periods (Plews et al., 2013). Indeed, parasympathetic activity has been utilized to personalize recovery periods between sessions in endurance training (Herzig et al., 2018; Duking et al., 2021). Previous studies aiming to compare endurance training protocols with individualized vs. fixed recovery prescriptions observed greater adaptations in maximum running speed (favoring the individualized group) (Kiviniemi et al., 2007), even when performing a reduced total number of sessions throughout the intervention period (Vesterinen et al., 2016). Although the use of this tool is accepted and used for individualized prescription of endurance training, little is known about its efficacy in prescribing RT.

To the best of our knowledge, only one study has compared the effects of individualized RT prescription with a predefined RT protocol. De Oliveira et al. (2019) investigated whether a RT program, conducted with individualized autonomic recovery between sessions (IND) using RMSSD as a recovery parameter, demonstrated that IND sessions promoted greater gains in strength and muscle mass and reduced the variability of these adaptations compared to a RT program with fixed recovery intervals (FIX) in young untrained men. The results revealed no significant difference in strength gains, muscle hypertrophy, or variability of adaptive responses between the protocols. Notably, the FIX group performed a training frequency of three sessions per week, resulting in a 48–72 h interval between sessions. This regimen likely ensured sufficient recovery for most participants, despite the absence of individualized recovery protocols. However, these findings cannot be generalized to older individuals, as parasympathetic activity (e.g., RMSSD values), tends to decrease with aging (Voss et al., 2015; Choi et al., 2020). Although the impact of aging on RT prescription through HRV is not fully established, aging is associated with increased arterial stiffness and elevated systolic blood pressure, which can subsequently influence HRV (Paneni et al., 2017). For example, following a RT session, parasympathetic activity in older adults may take longer to return to baseline, indicating a slower recovery compared to younger individuals (Sandercock et al., 2005). Therefore, it may be plausible to suggest that older individuals may requires longer recovery periods.

Considering the possibility of individualizing recovery between RT sessions using HRV, through the RMSSD parameter, the aim of this study was to investigate whether older adults with IND would experience greater adaptations in muscle strength, muscle mass, functional performance and reduce the variability of these adaptations compared to FIX. Moreover, we aimed to examine the effects of these RT protocols on volume load. We hypothesized that the IND protocol would result in greater increases in muscle strength, hypertrophy, functional performance and volume load accompanied by reduced variability in these adaptations, compared to the FIX program.

## Methods

### Participants

Twenty-one women were recruited for the study, with an average age of 66 ± 5 years, body mass of 71.3 ± 14.7 kg, and height of 160.2 ± 6.7 cm. Inclusion criteria required participants to be non-smokers and free from any diagnosed diseases or conditions that could interfere with autonomic function or the study outcomes. Additionally, participants were required to maintain a stable lifestyle, with no engagement in resistance training or other structured exercise programs in the 6 months prior to the study. Exclusion criteria included joint or muscle injuries, use of medications such as anti-inflammatory drugs, analgesics, antidepressants, or central nervous system depressants that could affect heart rate variability (HRV), and any cardiovascular, respiratory, renal, musculoskeletal, or metabolic diagnoses (e.g., diabetes mellitus). Participants with uncontrolled hypertension (SBP ≥ 140 mmHg, DBP ≥ 90 mmHg) were also excluded. Furthermore, participants were instructed to abstain from caffeine consumption during the experimental period. The study was conducted according to the Declaration of Helsinki and ethical approval was granted by the ethics committee (#62381316.2.0000.5504) at the local University.

### Study design

In the first week, all participants underwent HRV measurement for five consecutive days to determine baseline RMSSD values, which was used as an individualized recovery parameter between RT sessions. Following the 5-day period, participants underwent measurements of the muscle cross-sectional area (CSA), followed by the dynamic maximal strength test (one-repetition maximum [1RM]). Subsequently, rate of force development (RFD) and isometric peak torque (PT) were performed along with all the functional performance tests (chair stand [CS], timed up and go [TUG], 6-min walking [6MW] and maximal gait speed [MGS]). Then, participants were randomly allocated in one of the following experimental conditions: IND (n = 11) or FIX (n = 10) All participants underwent a period of 7 weeks of RT. Ninety-six hours after the last training session, all tests were repeated in the same order and interval as baseline measurements.

### Heart rate variability

All HRV measurements were consistently conducted at the same time of day for each participant over a period of five consecutive days (from Monday to Friday). The average value obtained from these measurements was used to calculate the individual RMSSD baseline. During the recording week, participants were instructed to consume light meals and prioritize sufficient rest. The R-R interval recordings were obtained using the Polar^®^ S810i (Polar Vantage, Finland) heart rate monitor, recognized as a valid and reliable method for such measurements (Gamelin et al., 2006). Participants were instructed to lie down in a supine position in a pre-designated room and were advised not to sleep, move, or talk during the measurements. The recording session lasted for 10 min, with a sampling rate of 1,000 Hz.

The collected data were subsequently analyzed using Kubios HRV software (Version 2.2, Biosignal Analysis and Medical Imaging Group, Kuopio, Finland), which has been validated for reliability and accuracy in HRV analysis (Tarvainen et al., 2014; Perrotta et al., 2017). Initial data processing involved artifact correction through the software’s built-in automatic method, utilizing the cubic spline interpolation technique (Lipponen and Tarvainen, 2019). Following artifact correction, analyses were conducted by the same evaluator using the full 10-min recording period. To determine baseline RMSSD values, we adopted the values for the RMSSD baseline for each participant, as the mean minus one standard deviation, since this arbitrary value may be a small variation with respect to observed over the 5 days of measurement. These baseline values served as an index of recovery from training sessions, as outlined by De Oliveira et al. (2019).

### Muscle cross-sectional area

The CSA of the vastus lateralis was obtained by the ultrasound. Using a metric tape graduated in centimeters, an experienced researcher delineated the measurement between the lateral femoral epicondyle and the greater trochanter. Subsequently, the collection point was ascertained, equating to 50% of said measurement. An adhesive Velcro strip was affixed to the ankle region, thereby immobilizing one limb to the other and prevent movement during the collection procedure. To precisely guide the trajectory of the linear B-mode ultrasound probe, the skin was demarcated transversely with 2 cm intervals from medially to laterally. A B-mode ultrasound apparatus, adjusted with a 7.5 MHz linear probe (Samsung, MySono U6, São Paulo, Brazil), was employed for image acquisition. Throughout the entire employment of the linear probe, transmission gel was administered to ensure acoustic coupling and, in addition, to prevent epidermal compression. Images were collected at 2 cm intervals, in adherence to the prior stipulated markings (Lixandrao et al., 2014). The Power Point software (Microsoft, United States) was used for the reconstruction of the CSA. The area value was computed through computerized planimetry using the ImageJ Software (Lixandrao et al., 2014).

### Maximal dynamic strength test

The maximal dynamic strength was measured through the one-repetition maximum (1RM) test on the knee extension (1RM_LE_) and vertical bench press (1RM_BP_) exercises (Brown and Weir, 2001). Initially, participants performed a general warm-up on a cycle ergometer at a speed of 20 km.h−^1^ for 5 minutes, followed by two sets of specific warm-ups performed on knee extension and vertical bench press machines. The first set consisted of eight repetitions at 50% of the estimated 1RM, followed by a set of three repetitions at 70% of the estimated 1RM, with a 2-min rest between warm-up sets. Afterwards, participants had up to five attempts to reach their 1RM in each exercise, with a 3-min rest between attempts. The highest load lifted with a proper form (i.e., full range of motion, individually set for each participant) was considered the 1RM.

### Isometric peak torque (PT) and rate of force development (RFD)

The PT and RFD were measured using an isokinetic dynamometer (Biodex System 3; Biodex Medical Systems, Shirley, NY, United States). Prior to the test, individuals performed a 5-min warm-up on a cycle ergometer at 60 rpm and 25 w of power. Then, participants seated on the dynamometer chair, with their torso and right leg stabilized by straps. The knee and dynamometer center of rotations were aligned, and the distance of the dynamometer attachment was adjusted to ensure that the ankle was in a comfortable position. The knee angle was set at 60°, and specific warm-up was performed, consisting of 10 submaximal, 2-s long voluntary isometric contractions, separated by a 20-s interval between each contraction. The number of repetitions used for the specific warm-up was set to allow for familiarization with the protocol (Libardi et al., 2016). After completing the warm-up, four maximal voluntary isometric contractions were performed with 3 min of rest between each attempt. Participants were instructed to produce torque as fast as possible and to maintain the maximal torque reached for 2 s, and then to relax as quickly as possible at the end of the force maintenance time. The torque production curve should follow a “rectangular pattern” (i.e., the fastest possible torque growth, maximum torque maintenance, and the fastest possible torque decay). Whenever the pattern differed from this, the attempt was discarded. Strong verbal encouragement was given in all attempts. Finally, the RFD was calculated using the formula ∆*T* · ∆t^−1^ (Libardi et al., 2016), through a routine written in MATLAB language (version 7.0 - Math Works Inc.) and the PT was considered as the higher value obtained within the four attempts.

### Functional performance tests

#### Chair stand (CS)

The CS test started with participants seated on a 43 cm-high chair with their hips and back fully supported on the backrest, knees flexed at 90°, and feet positioned on a force platform (AccuGait, Advance Mechanical Technology Inc. - AMTI, Boston, United States). Participants were instructed to cross their arms in front of their chest with their hands touching the opposite shoulders. Instructions consisted of standing up until reaching total upright position, and sit down until their back touched the backrest of the chair and repeat this movement five times as quickly as possible with feet in contact with the force platform (Jones et al., 1999). Time to complete this task was recorded through the Balance Clinic software (AMTI, Boston, United States) and analyzed using a routine written in MATLAB language (version 7.0 - Math Works Inc.).

#### Timed up and go (TUG)

Participants began the TUG seated on a 43 cm high chair with side supports, and feet fully in contact on a force platform. They were then instructed to stand up with the help of the side supports, walk a distance of 3 m and return to sit down in the chair as quickly as possible, without changing from walking to running pattern (Podsiadlo and Richardson, 1991). Time to complete this task was measured and analyzed as described for the CS test.

#### 6-minutes walking (6MW)

The 6MW test was conducted on a flat surface, demarcated by marks over a distance of 95 m, with the evaluator recording the distance covered in 6 min (Pasma et al., 2014). Participants were verbally instructed and encouraged to walk as fast as possible without running. If participants experienced any discomfort or fatigue, they were allowed to voluntarily stop the test.

#### Maximal gait speed (MGS)

For MGS, participants were instructed to cover 15 m twice at maximum walking speed, without transitioning to a running pattern. Attempts were split by 1 min of rest. The time were recorded using a photocell (Speed Test Fit, Cefise Biotecnologia Esportiva, São Paulo, Brazil). The first and last 2.5 m were not included in the measurements because they were considered periods of acceleration and deceleration, respectively. The results were obtained by averaging the two attempts (Pasma et al., 2014).

### Resistance training protocol

Prior to each RT session, RMSSD values were used to determine whether participants in the IND group had recovered from the previous session. Participants were considered recovered and ready for a training session if their RMSSD values were equal to or higher than their individual baseline, which was defined as the mean minus one standard deviation of the RMSSD values measured over five consecutive days during the familiarization period. Conversely, any RMSSD value below this threshold was interpreted as insufficient recovery, and participants were instructed to abstain from training and return to the laboratory 24 h later, except on Fridays, when the next training session would be on Monday. The FIX group performed RT sessions every 48 h on a fixed schedule (i.e., Monday, Wednesday, and Friday), regardless of whether the RMSSD values were above or below the baseline. Weekends were considered recovery days for both groups.

The RT protocol consisted of eight exercises performed in the following order for both groups: knee extension, 45° leg press, knee flexion, bench press, front lat-pull down, triceps extension, biceps curl, and shoulder press. Three sets of 9–12 repetition maximum (RM) at ∼80% of 1RM until concentric failure were performed for each exercise. Exercises were interrupted if participants were unable to maintain the predetermined and considered adequate range of motion. The load was increased whenever participants performed more than 12 RM and reduced when the numbers of repetitions was less than 9 RM, in order to maintain the number of repetitions in the desired range of motion. A 2-min rest interval was adopted between sets and exercises. Volume load (sets × repetitions × load [kg]) was expressed as the sum of the volume load in each training session, considering all exercises.

### Statistical analyses

An unpaired t-test was applied to compare the baseline values of all dependent variables and the volume load between the two groups. A mixed-model analysis for repeated measures was performed with groups (IND and FIX) and time (Pre and Post) as fixed factors, and participants as a random factor for CSA, 1RM_LE_, 1RM_BP_, PT, RFD, CS, TUG, 6MW, MGS. In case of significant *F*-values, a Tukey adjustment was used for pairwise comparisons. Significance was established as *P* < 0.05. Additionally, the effect sizes (ES) and respective confidence intervals (CI) of the differences between the delta change (Post–Pre) in each group were calculated according to Hedges and Olkin (1985). Positive and negative CIs not crossing zero (0) were considered significant (Nakagawa and Cuthill, 2007). Finally, to assess the homogeneity of variances across groups, Levene’s test was conducted on each dependent variable.

## Results

### Reproducibility

The coefficient of variation based on the typical error was calculated from two assessments with a 72-h interval between them. The values are: 1.27% for CSA, 10.49% for 1RM_LE_, 5.01% for 1RM_BP_, 3.84% for CS, 3.69% for TUG, 3.80% for 6MW, 3.53% for MGS.

### Baseline values

There were no significant differences between groups in baseline values for all variables investigated (*P* > 0.05).

### Individualized recovery

**FIGURE 1:**
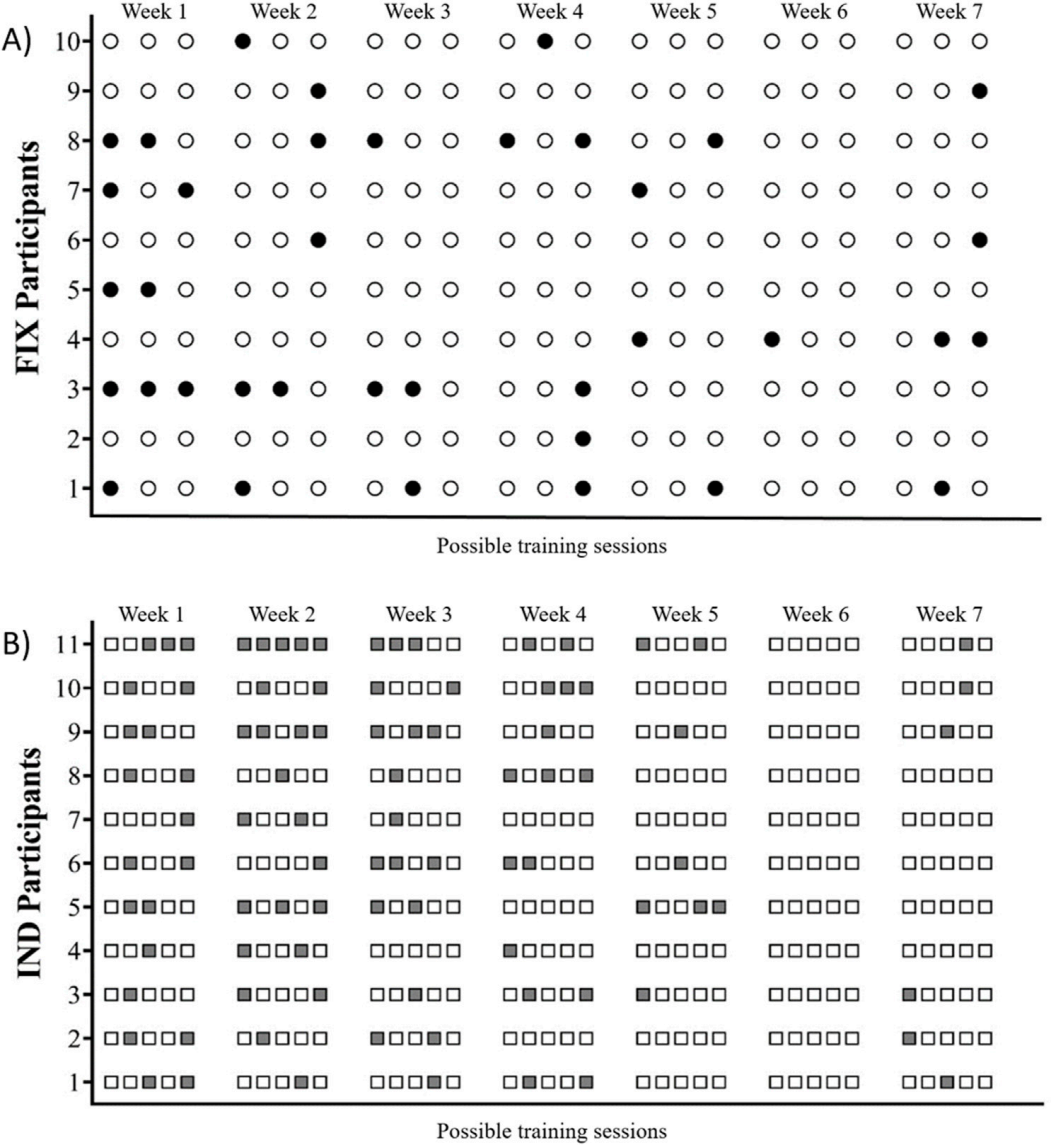
**(A)** Circles represent training sessions performed by participants in the FIX group. Black-filled circles indicate sessions where RMSSD values had not returned to baseline (non-recovered state), while white circles represent sessions conducted in a recovered state. **(B)** Gray squares indicate days when participants in the IND group abstained from training due to RMSSD values not returning to baseline (non-recovered state), whereas white squares represent training sessions.

Individually, the subjects’ responses varied significantly. For the IND group, individuals who completed the highest number of training sessions managed to finish 31 out of the possible 35 sessions, while the one who completed the fewest sessions only did 19 (a smaller quantity than the 21 sessions carried out by members of the FIX group). When examining the FIX group, the individual who showed the most recovery, completed only one training session without returning to baseline RMSSD values. Conversely, the individual who showed the least recovery during the intervention underwent eight training sessions without recovering. The individual recovery and training responses of each subject in both groups are detailed in Figure 1.

### Training frequency and volume load

The IND group performed an average of four sessions per week, totaling 27 sessions. A group vs. time interaction was demonstrated for weekly frequency (*F*_[6, 123]_ = 8.83, *P* < 0.0001). In contrast, the FIX group maintained a frequency of three weekly sessions over the 7 weeks, totaling 21 training sessions. Post hoc analysis revealed higher training frequency in weeks 5 (4.3x/week), 6 (5x/week), and 7 (4.5/week) compared to weeks 1 (3.2/week) (*P* < 0.001 for all), 2 (2.8/week) (*p* < 0.001 for all) and 3 (3.3/week) (*P* < 0.01 for all). Additionally, weeks 6 and 7 showed higher training frequency compared to week 4 (3.5/week) (*P* < 0.01 for all). For the between-groups comparison, training frequency in weeks 5, 6 and 7 was significantly higher for the IND group compared to the FIX group (*P* < 0.001 for all). Regarding volume load, the IND group showed a higher accumulated volume load over the 7-week training period compared to the FIX group (120833.50 ± 20683.52 kg and 159174.09 ± 42836.52 kg, respectively; *P* = 0.01).

### Muscle cross-sectional area (CSA)

The mixed model analysis revealed significant main effect of time for CSA (*F*_[1, 21]_ = 106.18, *P* < 0.0001), with no significant effect of group (*F*_[1, 21]_ = 0.37, *P* = 0.554) or group vs. time interaction (*F*_[1, 21]_ = 0.34, *P* = 0.573). The 95% CI of ES analysis revelated no significant differences between groups for changes in CSA (Table 1).

**TABLE 1:** Muscle cross-sectional area and muscular strength outcomes following fixed (FIX) and individualized (IND) recovery between resistance training sessions.

Variables	FIX			IND			ES (95% CI)
	Pre	Post	Δ (%)	Pre	Post	Δ (%)	Δ FIX vs. Δ IND
CSA (cm^2^)	14.27 ± 3.10	15.62 ± 3.72*	9.46	13.38 ± 3.15	14.57 ± 3.21*	8.89	−0.06 (−0.92 to 0.79)
1RM_LE_ (kg)	18.60 ± 3.69	27.30 ± 5.12*	46.77	19.73 ± 5.64	29.09 ± 7.03*	47.44	0.15 (−0.70 to 1.01)
1RM_BP_ (kg)	27.10 ± 6.57	37.70 ± 8.68*	39.11	27.73 ± 6.47	37.55 ± 7.93*	35.41	0.42 (−0.44 to 1.29)
PT (N/m)	115.15 ± 20.98	121.96 ± 19.76*	5.91	126.53 ± 27.62	152.35 ± 54.24*	20.41	0.63 (−0.27 to 1.48)
RFD (N.m.s^-1^)	372.85 ± 172.39	387.27 ± 169.83	3.86	396.27 ± 97.89	437.48 ± 124.22	10.40	0.22 (−0.64 to 1.08)

Abbreviations and symbols: CSA, vastus lateralis muscle cross-sectional area; 1RM_LE_, leg extension one-maximum repetition; 1RM_BP_, bench press one-maximum repetition; PT, peak torque; RFD, rate of force development; Pre, baseline values; Post, values after training; Δ (%), Changes from pre to post training; ES (95% CI), effect size of the differences between groups and its confidence interval. *Significantly different from Pre (main time effect *P* < 0.05).

### Maximal dynamic strength (1RM)

There was no significant group vs. time interaction (*F*_[1, 21]_ = 0.48, *P* = 0.50) or group main effect (*F*_[1, 21]_ = 0.34, *P* = 0.56) for 1RM_LE_ or 1RM_BP_ However, a main effect of time was observed (*F*_[1, 21]_ = 189.78, *P* < 0.0001). Similarly, a main effect of time (*F*_[1, 21]_ = 295.54, *P* < 0.0001), but not a main effect of group (*F*_[1, 21]_ = 0.14, *P* = 0.71) or a group vs. time interaction (*F*_[1, 21]_ = 0.94, *P* = 0.35) was demonstrated for 1RM_BP_. The 95% CI of ES analysis revelated no significant differences between groups for changes in 1RM_LE_ and 1RM_BP_ (Table 1).

### Isometric peak torque (PT) and rate of force development (RFD)

For PT, a significant main time effect was observed (*F*_[1, 21]_ = 5.48, *P* = 0.03), without a significant main effect of group (*F*_[1, 21]_ = 1.26, *P* = 0.28) or group vs. time interaction (*F*_[1, 21]_ = 0.86, *P* = 0.37). Furthermore, RFD showed no significant effect of time (*F*_[1, 21]_ = 1.06, *P* = 0.31), group (*F*_[1, 21]_ = 0.91, *P* = 0.34), or group vs. time interaction (*F*_[1, 21]_ = 0.23, *P* = 0.6389). The 95% CI of ES analysis revelated no significant differences between groups for changes in PT and RFD (Table 1).

### Functional performance

The functional performance results are presented in Table 2. The mixed model analysis revealed a significant main effect of time for CS (*F*_[1, 21]_ = 46.07, *P* < 0.0001), with no significant effect of group (*F*_[1, 21]_ = 1.20, *P* = 0.28) or group vs. time interaction (*F*_[1, 21]_ = 0.99, *P* = 0.32). Similarly, TUG presented a main time effect (*F*_[1, 21]_ = 24.17, *P* = 0.0004), with no significant effect of group (*F*_[1, 21]_ = 0.27, *P* = 0.61) or group vs. time interaction (*F*_[1, 21]_ = 0.28, *P* = 0.60). Additionally, 6 MW showed a significant main effect of time (*F*_[1, 21]_ = 8.32, *P* = 0.007), with no significant effect of group (*F*_[1, 21]_ = 0.04, *P* = 0.85) or group vs. time interaction (*F*_[1, 21]_ = 1.58, *P* = 0.21). Lastly, MGS also showed a main effect of time (*F*_[1, 21]_ = 9.60, *P* = 0.009), with no significant effect of group (*F*_[1, 21]_ = 0.95, *P* = 0.34) or group vs. time interaction (*F*_[1, 21]_ = 0.03, *P* = 0.87). The 95% CI of ES analysis revelated no significant differences between groups for changes in functional performance.

**TABLE 2:** Functional performance outcomes following fixed (FIX) and individualized (IND) recovery between resistance training sessions.

Variables	FIX			IND			ES (95% CI)
	Pre	Post	Δ (%)	Pre	Post	Δ (%)	Δ FIX vs. Δ IND
CS (s)	13.76 ± 3.28	9.55 ± 1.42*	−30.60	12.93 ± 1.55	9.59 ± 1.45*	−25.83	0.42 (−0.51 to 1.26)
TUG (s)	8.95 ± 1.68	7.46 ± 0.84*	−16.64	8.47 ± 1.53	7.28 ± 0.94*	−14.05	0.20 (−0.66 to 1.06)
6MW (m)	589.79 ± 55.65	609.47 ± 75.96*	3.33	572.21 ± 63.29	622.35 ± 46.01*	8.76	0.70 (−0.18 to 1.58)
MGS (m/s)	1.92 ± 0.23	2.00 ± 0.14*	4.16	2.04 ± 0.25	2.15 ± 0.24*	5.39	0.10 (−0.76 to 0.96)

Abbreviations and symbols: CS, chair stand; TUG, timed up and go; 6MW, 6-min walking; MGS, maximum gait speed; Pre, baseline values; Post, values after training; Δ (%), Changes from pre to post training; ES (95% CI), effect size of the differences between groups and its confidence interval. *Significantly different from Pre (main time effect, *P* < 0.05).

### Variability in adaptations to resistance training

Levene’s test found no differences in the variability of responses for CSA (*P* = 0.325), 1RM_LE_ (*P* = 0.675), 1RM_BP_ (*P* = 0.238), PT (*P* = 0.516), RFD (*P* = 0.610), CS (*P* = 0.687), TUG (*P* = 0.478), 6 MW (*P* = 0.521), and MGS (*P* = 0.312). The values of CSA ranged from (IND: 4.63%–13.11%; FIX: 1.34%–16.74%), 1RM_LE_ (IND: 20.00%–66.67%; FIX: 29.17%–66.67%), 1RM_BP_ (IND: 25.00%–75.00%; FIX: 30.00%–55.56%), PT (IND: −12.42%–89.80%; FIX: −18.75%–30.12%), RFD (IND: −48.02%–80.23%; FIX: −45.38%–48.72%), CS (IND: 10.93% to −47.17%; FIX: −3.22% to −48.82%), TUG (IND: 2.31% to −26.76%; FIX: −4.08% to −35.52%), 6 MW (IND: −3.73%–27.52%; FIX: −14.22%–11.47%), and MGS (IND: −10.56%–20.05%; FIX: −5.26%–15.31%).

## Discussion

The aim of the present study was to compare whether the individualization of training (IND) based on the RMSSD parameter, measured before each training session, would result in greater morphological and functional adaptations compared to fixed recovery training (FIX) in older women. Our main finding revealed that individualizing recovery through the RMSSD did not result in greater adaptations or decrease the variability of adaptive responses, despite the IND group performing a higher training frequency and volume load in older women.

Although parasympathetic activity, measured through the RMSSD parameter, is widely used to monitor and prescribe endurance exercises (Plews et al., 2013; Schmitt et al., 2015; Vesterinen et al., 2016; da Silva et al., 2019), little is known about its effectiveness in RT prescription. Contrary to our initial suggestion that older individuals would require a longer autonomic recovery period between RT sessions, the IND group showed shorter autonomic recovery periods compared to the FIX group, resulting in a higher training frequency. Indeed, our findings revealed a significant increase in weekly training frequency from the fifth week of training for the IND group, resulting in shorter intervals between training sessions (∼24 h between sessions) compared to the FIX group. Consequently, the IND group completed an average of 27 training sessions over the seven-week experimental period, compared to 21 sessions for the FIX group. While speculative, this phenomenon may be partially attributed to the gradual reduction in cardiovascular stress induced by the training sessions over the weeks, leading to smaller changes in RMSSD values. However, this hypothesis should be tested in future studies.

Curiously, despite the IND group demonstrated a higher training frequency from the 5th week onwards (>4x/week), leading to a greater volume load, there were no significant differences in 1RM, PT and CSA increases compared to the FIX group. Our findings align with Barcelos et al. (2018), who observed no significant differences in muscle strength gains and hypertrophy between lower (2–3 weekly sessions) and higher (5 weekly sessions) RT frequencies in untrained young individuals. In older adults, while no study has investigated protocols with higher training frequencies, a recent meta-analysis included studies examining RT frequencies of one, two, or three times per week, and also found no significant effect of weekly frequency on muscle hypertrophy outcomes (Kneffel et al., 2021). Furthermore, muscle strength results revealed that frequencies greater than twice a week do not promote additional gains for this population. Taken together, it is plausible to suggest that increasing weekly frequency during the initial phases of training may not be necessary to maximize muscle strength gains and hypertrophy in older women. Conversely, adopting a higher training frequency does not seem to impair muscle strength and mass gains for this population, at least when participants adequately recover between training sessions.

Regarding RFD, no significant increase was observed in either group. Although somewhat challenging to explain, our results for RFD may be partially justified by the RT protocol adopted. During all training sets, participants were instructed to perform repetitions to concentric failure, which markedly reduces the contraction velocity and power output at the end of the sets (Sánchez-Medina and González-Badillo, 2011; Vieira et al., 2021). Therefore, it is possible that the protocol adopted in our study was not designed to induce significant improvements in RFD. Indeed, previous studies have noted that both intentional and unintentional low-velocity movements during RT may have influenced our RFD findings (Bergamasco et al., 2022).

For functional performance, improvements were observed in all applied tests (i.e., TUG, CS, 6MW, and MGS), with no significant differences between protocols. These findings are consistent with previous studies, which found no significant differences in functional performance when comparing one, two, or three weekly RT session (Taaffe et al., 1999; Turpela et al., 2017). To our knowledge, only the study by Farinatti et al. (2013) observed that higher RT frequencies promote greater improvements in functional performance (three vs. one or two sessions per week). These differences may be partially explained by the characteristics of the RT protocols. While in our study, participants were instructed to perform all sets to the point of concentric muscular failure within a 9–12 RM range, the majority of the exercises performed in the protocol implemented by Farinatti et al. (2013) were comfortably performed (far from concentric failure). Therefore, one can suggest that once training sets are performed with a high-level of effort, no additional benefits on functional performance emerge from increasing RT-frequency. This study is not without its limitations. The first important consideration is that the results observed are confined to a seven-week training period. Prolonged exposure to a relatively high training frequency, as observed in the IND group, coupled with an extended training duration, could have resulted in adverse effects on morphological and functional adaptations in certain individuals. Another limitation is the absence of metabolic measurements, such as inflammatory markers and indicators of muscle damage, which constrains our ability to determine whether the high training frequency exerted any negative physiological effects. However, one of the primary consequences of muscle damage is impaired force generation and exercise performance, outcomes that were not evident in the participants. Regardless of the group, both demonstrated an increased volume load, particularly the group that underwent individualized autonomic recovery. Therefore, it is improbable that non-functional overreaching or overtraining occurred within the scope of this intervention. Finally, another limitation of this study is the relatively small sample size, which may have resulted in low statistical power. Future studies with larger samples are recommended to confirm these findings and provide further insights.

## Conclusion

Individualizing autonomic recovery periods between RT sessions using RMSSD parameters does not promote to greater improvements in muscle strength, muscle mass, or functional performance, nor does it reduce the variability of adaptive responses, compared to a fixed recovery RT protocol in healthy older women.

## Data Availability

The raw data supporting the conclusions of this article will be made available by the authors, without undue reservation.
